# A taxonomic study of Chinese species of the *alberti* group of *Metaphycus* (Hymenoptera, Encyrtidae)

**DOI:** 10.3897/zookeys.285.4142

**Published:** 2013-04-05

**Authors:** Ying Wang, Cheng-De Li, Yan-Zhou Zhang

**Affiliations:** 1 Division of Forest Protection, School of Forestry, Northeast Forestry University, Harbin, 150040, China; 2 Key Laboratory of Zoological Systematics and Evolution, Institute of Zoology, Chinese Academy of Sciences, Beijing 100101, China

**Keywords:** Chalcidoidea, parasitoids, natural enemy, new species, China

## Abstract

Ten *alberti*-group species of the genus *Metaphycus* Mercet from China are reviewed. Six species *Metaphycus dorsalis* **sp. n.**, *Metaphycus chinensis* **sp. n.**, *Metaphycus wui* **sp. n.**, *Metaphycus stylatus* **sp. n.**, *Metaphycus fusiscapus* **sp. n.** and *Metaphycus fusiformis* **sp. n.** are described as new to science. Four known species from China are redescribed. A key to the females of the Chinese species is given and photomicrographs are provided to illustrate morphological characters of these species. All specimens unless otherwise specified are deposited in the National Zoological Museum of China Institute of Zoology Chinese Academy of Sciences Beijing.

## Introduction

*Metaphycus* is a large genus of the family Encyrtidae, including 455 species worldwide (Noyes 2012). All species of *Metaphycus* with known biology are primary endoparasitoids of scale insects, mainly species of Coccidae, Diaspididae, Eriococcidae and Margarodidae (Guerrieri and Noyes 2000; DeBach and Rosen 1991). *Metaphycus* play a role in the control of agricultural and forestry pests, and probably contribute to the population control of potential pests of forest and fruit trees, ornamentals and agricultural crops. In China, *Metaphycus parasaissetiae* controls their host *Parasaissetia nigra* at the earlier stage of the egg-laying season (Zhang et al. 2010). It is one of the most successful groups of insects to have been used in the biological control of scale insects (such as *Coccus* and *Saissetia*) (Guerrieri and Noyes 2000; Noyes 2004; Lotfalizadeh 2010).

Due to the economical and particularly the extraordinary diversification of *Metaphycus*, many taxonomic works have been published and a few good keys have been presented for the regional species of *Metaphycus* (Compere 1940; Annecke and Mynhardt 1971, 1972, 1981; Myartseva 1987; Viggiani and Guerrieri 1988; Guerrieri and Noyes 2000; Trjapitzin 1989; Zeya and Hayat 1993). Most of them are based on the distinction of species groups using the palpal formula as suggested by Compere and Annecke (1960). Based on the palpal formula, Compere and Annecke (1960) suggested dividing the genus *Metaphycus* into three species groups: *alberti*-group (Plate I–A) (with 2-segmented maxillary palpi), *insidiosus*-group (Plate I–B) (with 3-segmented maxillary palpi) and *zebratus*-group (Plate I–C) (with 4-segmented maxillary palpi). The *alberti*-group is interpreted here as having 2-segmented maxillary and 2-segmented labial palpi (Anneck and Mynhardt 1971; Guerrieri and Noyes 2000). Graham (1959) used *hederaceus* as the group name, but later he proposed *asterolecanii* for the same group, since *hederaceus* belongs to *Aphycus* rather than *Metaphycus* (Graham 1969). Tachikawa (1963) was the first author to use *alberti* as the name of this group, and this is widely accepted (Guerrieri and Noyes 2000; Noyes 2004; Zeya and Hayat 1993). Guerrieri and Noyes (2000) described *Metaphycus babas* as a new species, with a palpal formula of 2-3. Therefore, they prefer to define these species on the number of segments in the maxillary palpi alone. Noyes (2004) broke with this framework and introduced several other characters (e.g. presence or absence of subapical setae on the 2^nd^ valvifer). These characters are very difficult to observe unless high quality slide-mounted specimens are prepared. In the course of this work, the framework of Compere and Annecke (1960) was followed.

The Chinese fauna of *Metaphycus* is poorly known, though some taxonomic contributions (Jiang 1982; Shi 1986; Xu and Jing 1990) have been made in the later years of past century. But some synonyms and homonyms can be found easily, such as *Metaphycus ericeri* Xu & Jiang 1990 (renamed *Metaphycus xujiangi* by Özdikmen 2011). Recently, several new species and new records have been reported from China (Dang and Wang 2002; Li and Xu 2006; Li and Li 2008; Zhang and Huang 2006; Zhang and Wu 2008; Tan 2008). So far, more than twenty species of *Metaphycus* species have been recorded from China, including four *alberti* group species. To facilitate the accurate identification of this large group of Encyrtidae, systematic study of all species known in China is necessary (Zhang and Wu 2008). The present work is part of this effort.

Accurate identification of *Metaphycus* species is very difficult because of their small size and general appearance (Annecke and Mynhardt 1971; Guerrieri and Noyes 2000). Thus high quality slide preparation is needed, and it is necessary to dissect the mouthparts and ovipositor parts. The characters (e.g. body coloration, width of frontovertex) used in the keys to species are disputable (Guerrieri and Noyes 2000); however our recent studies using molecular markers show these characters are arguable and can help us to disentangle these species complexes (unpublished data).

Morphological terminology and abbreviations follow those of Noyes (2004). Absolute measurements were used for body length. Relative measurements were used for other dimensions and measured with a Motic SMZ-168 stereomicroscope, under 50x magnification, and the absolute measurement of each unit is 0.02 mm. The following abbreviations are used in the text:

F1, F2, … FnFunicle segment number


AODLargest diameter of anterior ocellus


HWHead width measured in facial view


FVMinimum width of the frontovertex


FVLLength of frontovertex from occipital margin to top of antennal scrobes as seen in dorsal view


MSMalar space or the minimum distance between eye and mouth margin


POLThe minimum distance between the posterior ocelli


OCLThe minimum distance between the posterior ocellus and the occipital margin


AOLThe minimum distance between posterior ocellus and anterior ocellus


OOLThe minimum distance between the eye margin and the adjacent posterior ocellus


PODLargest diameter of posterior ocellus


ELThe maximum diameter of eye


EWThe minimum diameter of eye


SLThe length of the scape


SWThe maximum width of the scape


FWLLength of fore wing excluding the marginal fringe


FWWThe maximum width of the fore wing excluding the marginal fringe


HWLLength of hind wing, excluding the marginal fringe


HWWWidth of hind wing, measured at the widest point, excluding the marginal fringe


MTLength of the mid tibia


OLLength of ovipositor


GLLength of the gonostylus


BMNHNatural History Museum, London, UK


ICZNInternational Commission of Zoological Nomenclature


IZCASInstitute of Zoology, Chinese Academy of Sciences, Beijing, PR China


USNMUnited States National Museum, Washington, DC, USA


ZJUZhejiang University, Hangzhou, China


SCUSichuang University, Chengdu, China


KYUNKyoto University, Kyoto, Japan


### Taxonomy of *Metaphycus* Mercet

*Aenasioidea* Girault, 1911: 171. Type species: *Aenasioidea latiscapus* Girault, by original designation. Synonymy by Noyes and Woolley (1994: 1329). Suppressed: *Metaphycus* given precedence over *Aenasioidea* by the International Commission of Zoological Nomenclature (ICZN 1998).

*Metaphycus* Mercet, 1917: 138. Type species: *Aphycus zebratus* Mercet, by monotypy.

*Tyndarichoides* Girault, 1920: 189. Type species: *Tyndarichoides mexicanus* Girault, by monotypy. Synonymy by Noyes and Woolley 1994: 1329.

*Euaphycus* Mercet, 1921: 97. Type species: *Encyrtus hederaceus* Westwood, by subsequent designation of Mercet 1925: 23, as subgenus of *Aphycus* Mayr. Synonymy by Compere and Annecke 1960: 384. *Encyrtus hederaceus* Westwood was misidentified by Mercet; see Graham 1969: 224–225.

*Metaphycus* Mercet, 1925: 28. Generic status.

*Mercetiella* Dozier, 1926: 98. Type species: *Mercetiella reticulata* Dozier, by original designation. Synonymy by Trjapitzin and Gordh 1978: 636.

*Oaphycus* Giraiult, 1932: 5. Type species: *Aphycus sanguinithorax* Girault, by original designation. Synonymy by Noyes and Hayat 1984: 298.

*Erythraphycus* Compere, 1947: 7. Type species: *Erythraphycus argyrocomus* Compere, by original designation. Synonymy by Noyes and Woolley 1994: 1329.

*Melanphycus* Compere, 1947: 5. Type species: *Pseudococcobius fumipennis* Timberlake, by original designation. Synonymy by Noyes 1980: 212.

*Anaphycus* Sugonjaev, 1960: 372. Type species: *Aphycus nitens* Kurdjumov, by original designation. Synonymy by Trjapitzin 1971: 126.

*Mesaphycus* Sugonjaev, 1960: 370. Type species: *Aphycus picearum* Erdős, by original designation. Synonymy by Guerrieri and Noyes 2000: 148.

*Notoencyrtus* De Santis, 1964: 211. Type species: *Notoencyrtus guttofasciatus* De Santis, by original designation. Synonymy by Noyes 1980: 212.

*Xenaphycus* Sharkov & Voynovich, 1988: 826. Type species: *Paraphycus flavovarius* Mercet, by subsequent designation of Trjapitzin (1982: 38). Synonymy by Guerrieri and Noyes 2000: 148.

*Aenigmaphycus* Sharkov & Voynovich, 1988: 826. Type species: *Aenigmaphycus paluster* Sharkov & Voynovich, by monotypy. Synonymy by Guerrieri and Noyes 2000: 148.

**Diagnosis.** Length 0.7–1.8 mm; robust and squat species; body largely orange, yellow to brown or black (the latter at maximum shiny), never with metallic luster, antenna usually with black and white or yellow segments, fore wing hyaline or partially infuscate, legs mostly yellowish, sometimes tibiae with dark rings. Head with occipital margin sharp; mandible mostly broad with 3 short, subequal teeth. Pronotum short, mesoscutum wider than long, notaular lines variable in length from virtually absent to reaching about 0.7× across mesoscutum; fore wing generally about 2.1–2.7× as long as broad and with uniform setation, postmarginal vein very short, stigmal vein well developed, longer than marginal and postmarginal vein together; linea calva usually closed and interrupted in posterior third by a few setae. Female: antenna almost always 11-segmented (formula 1163: 1 scape, 1 pedicel, 6 funicle, 3 clava). Gaster with hypopygium reaching half way along gaster to more or less reaching its apex; ovipositor sheath free, in most cases not exserted or only slightly exserted in *Metaphycus stylatus* sp. n. Male: generally darker and with more uniform colour in respect to that of corresponding female. Antenna 9-segmented (formula 1161: 1 scape, 1 pedicel, 6 funicle, 1 clava).

### Key to *Metaphycus* species of *alberti-*group (females) from China

**Table:** 

1	Scape (Figs 39, 58) not distinctly flattened and expanded, about or more than 4× as long as broad	2
–	Scape (e.g. Figs 15, 63, 77) distinctly flattened and expanded, less than 3.5× as long as broad	3
2	Fore wing (Fig. 42) hyaline, without a small infuscate area beneath stigma vein; ovipositor sheath strongly exserted and about 0.4× as long as ovipositor (Fig. 47)	*Metaphycus stylatus* sp. n.
–	Fore wing (Fig. 62) hyaline, with a small infuscate area beneath stigma vein; ovipositor sheath clearly exserted but only about 0.25× as long as ovipositor (Fig. 61)	*Metaphycus nadius* (Walker, 1838)
3	Scape (Figs 1, 8, 15) 2.6–3.5× as long as broad	4
–	Scape (e.g. Figs 23, 48, 77) not more than 2.5× as long as broad	6
4	Scape (Fig. 8) with a completely pale yellow dorsal margin, usually 3× as long as broad	*Metaphycus alberti* (Howard, 1898)
–	Scape (Fig. 1) with dorsal margin not completely pale yellow	5
5	Mesoscutum and scutellum with a longitudinal dark brown strip medially (Plate I–D)	*Metaphycus dorsalis* sp. n.
–	Mesoscutum and scutellum without a dark brown longitudinal strip	*Metaphycus dispar* (Mercet, 1925)
6	Scape (Fig. 70) with completely pale yellow dorsal margin, usually 2.4× as long as broad	*Metaphycus fusiformis* sp. n.
–	Scape (e.g. Figs 23, 48, 77) without completely pale yellow dorsal margin	7
7	Ovipositor longer than mid tibiae	8
–	Ovipositor shorter than mid tibiae	9
8	Mid and hind tibiae with distinct dark brown brown marking (Fig. 67); scape (Fig. 63) about 2× as long as broad	*Metaphycus fusiscapus* sp. n.
–	Mid and hind tibiae immaculate (Fig. 36), at most with a fuscous spot near base of mid tibiae; scape (Fig. 30) 2.3× as long as broad	*Metaphycus wui* sp. n.
9	F1–F3 subquadrate, not distinctly transverse; clava clearly shorter than scape, about 0.6× as long as scape (Fig. 77)	*Metaphycus xujiangi* Özdikmen, 2011
–	F1–F3 distinctly transverse; clava about as long as scape (Figs 23, 48)	10
10	Fore wing 2.4× as long as broad (Fig. 53), ocelli forming an angle of about 50°; ovipositor (Fig. 57) about 5.4× as long as ovipositor sheath	*Metaphycus ericeri* Trjapitzin, 1967
–	Fore wing 2.7× as long as broad (Fig. 25), ocelli forming an angle of about 40°; ovipositor (Fig. 29) about 4.8× as long as ovipositor sheath	*Metaphycus chinensis* sp. n.

#### Metaphycus dorsalis sp. n.

urn:lsid:zoobank.org:act:486A67C0-EE88-4D7C-988A-47ABE6CB1712

http://species-id.net/wiki/Metaphycus_dorsalis

Plate 1D Figs 1–7

##### Holotype.

♀, China, Yunnan, Xishuangbanna: 2009.XI.9, Coll. G. Tang (IZCAS).

##### Paratypes.

2♀♀, the same as holotype; 2♀♀, 1♂, Sichuan, Chengdu, 1961.VII.1–5, Coll. D. X. Liao (IZCAS).

**Figures 1–7.**
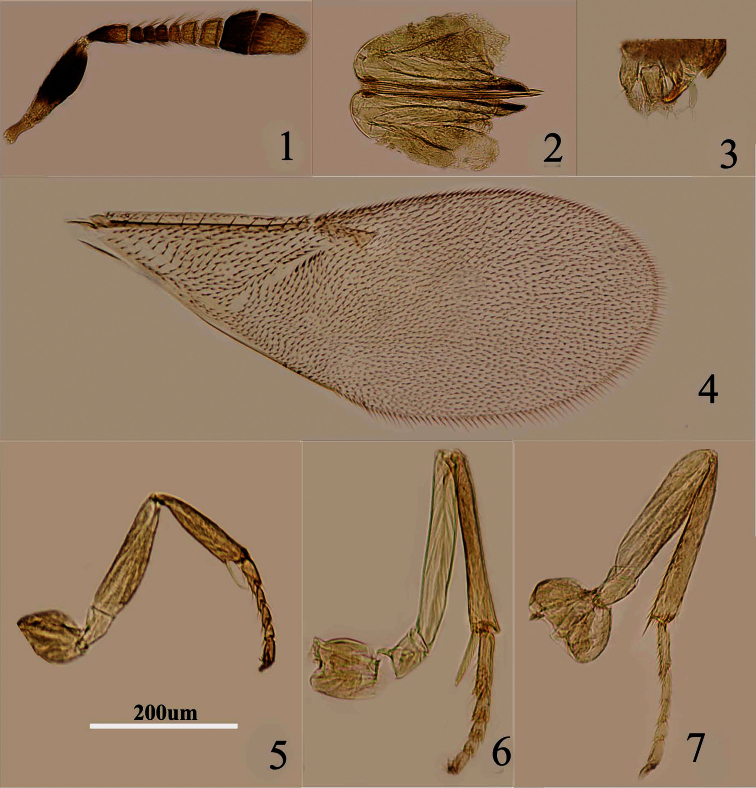
*Metaphycus dorsalis* sp. n. Female: **1** antenna **2** ovipositor **3** palpal formula **4** fore wing **5** fore leg **6** mid leg **7** hind leg.

Female: Body length, including ovipositor about 1mm. Frontovertex orange; orange in ocellar area, pale orange between occipital margin and posterior ocelli; immaculate with yellow from occiput to base of mandible; mouth margin medially yellow below torulus; rest of head, except occiput, white; antenna (Fig. 1) with radicle dark brown; scape with both faces dark brown, blackish, only base and apex white; pedicel dark brown in proximal one third, otherwise white; F1–F3 dark brown, F4 very pale brown, F5–F6 white, clava dark brown, becoming slightly paler towards apex, apex very pale brown; occiput with a brown area above foramen, rest white; neck of pronotum dark brown, posterior margin white, lateral spots relatively large and distinct; dorsum of thorax orange; sides and posterior margin of mesoscutum and axillae conspicuously bordered brown, mesoscutum and scutellum (Plate I–D) with brown line from front edge of mesoscutum to apex of scutellum; setae translucent pale brown, silvery in most lights; tegula white with apex pale brown; metanotum orange; mesopleuron yellow; prosternum and mesosternum pale yellow; legs (Figs 5–7) pale yellow; fore wing (Fig. 4) hyaline and with linea calva interrupted; venation yellow-brown; hind wing hyaline; propodeum medially orange-brown, laterally pale yellow; gaster orange and ovipositor sheath orange.

Head with polygonally reticulate sculpture and mesh size slightly less than that of one eye facet; frontovertex about one-fourth head width; ocelli forming an acute angle about 30°; eye not quite reaching occipital margin, separated by much less than diameter of a facet; frontovertex subparallel and from anterior ocellus slightly wider anteriorly; scrobes shallow and U-shaped; antenna with scape about 3–3.1× as long as broad; funicle with F1–F4 smallest, F5 a little larger than F4 but transverse, F6 largest and slightly wider than long; linear sensilla only on F5 and F6; clava 3-segmented, its apex more or less rounded but with a short slightly oblique truncation; mandible relatively broad with three subequal, apical teeth; palpal formula 2-2 (Fig. 3), notaular lines reaching about 0.4× across mesoscutum; fore wing venation and setation as in Fig. 4; ovipositor (Fig. 2) slightly exserted, about 5.6× as long as ovipositor sheath.

Relative measurements: HW 13, FV 3, FVL 7, POL 1.5, AOL 3.5, OOL 0.5, OCL 2, POD 1, AOD 1, EL 9, EW 6,MS 5, SL 6, SW 2, FWL 35, FWW 14, HWL 23, HWW 5, OL 10, GL 2, MT 10.

Male. Unknown.

##### Host.

Unknown.

##### Distribution.

China (Sichuan, Yunnan).

##### Etymology.

The specific epithet of this new species refers to the medial longitudinal dark brown strip on the mesoscutum and scutellum.

##### Diagnosis.

Antenna with scape about 3–3.1× as long as broad; mesoscutum and scutellum with brown line running from front edge of mesoscutum to apex of scutellum; legs pale yellow; fore wing hyaline and with linea calva interrupted. Using the keys of Trjapitzin (1989) and Guerrieri and Noyes (2000), this species runs to *Metaphycus dispar* (keys couplet 11 and 15). It can be separated from *dispar* as follows: mesoscutum and scutellum with a longitudinal dark brown strip in the middle (in *dispar*, mesoscutum and scutellum without a longtitudinal dark brown strip). Scape about 3× as long as broad (in *dispar*, scape about 3.3× as long as broad). Ovipositor about 5× as long as ovipositor sheath (in *dispar*, ovipositor about 4.3× as long as ovipositor sheath). The colour of the radicle is dark brown, and the metanotum is orange (in *dispar*, radicle with yellow, and metanotum is brown).

**Plate 1.**
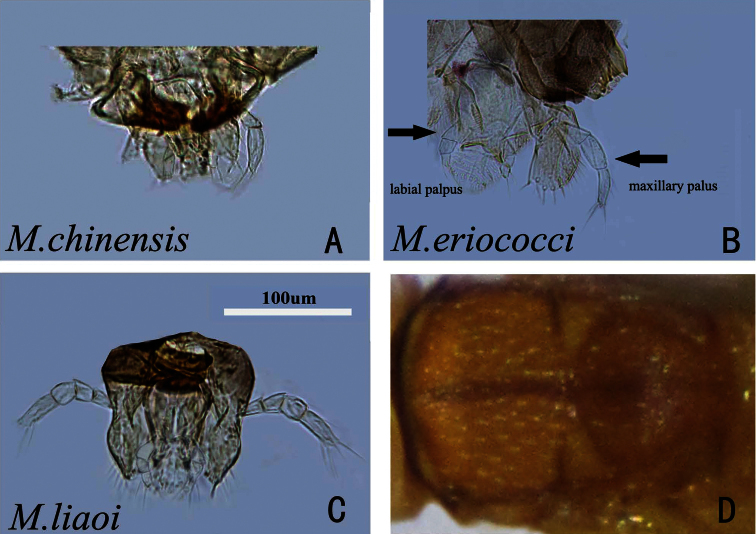
**A** palpal formula 2-2 (*Metaphycus chinensis* sp. n.) **B** palpal formula 3-3 (*Metaphycus eriococci*) **C** palpal formula 4-3 (*Metaphycus liaoi*) **D** thorax of *Metaphycus dorsalis* sp. n. in dorsal view.

#### Metaphycus alberti

(Howard, 1898)

http://species-id.net/wiki/Metaphycus_alberti

Figs 8–14

Aphycus alberti Howard, 1898: 247. Syntypes ♀♂, Australia (New South Wales), USNM, examined (part).
Metaphycus alberti (Howard); Compere 1957: 222, 224.
Metaphycus aurantiacus Annecke & Mynhardt 1981: 60–61. Synonymized with *alberti* by Noyes 2004: 254.

##### Female.

**Figures 8–14.**
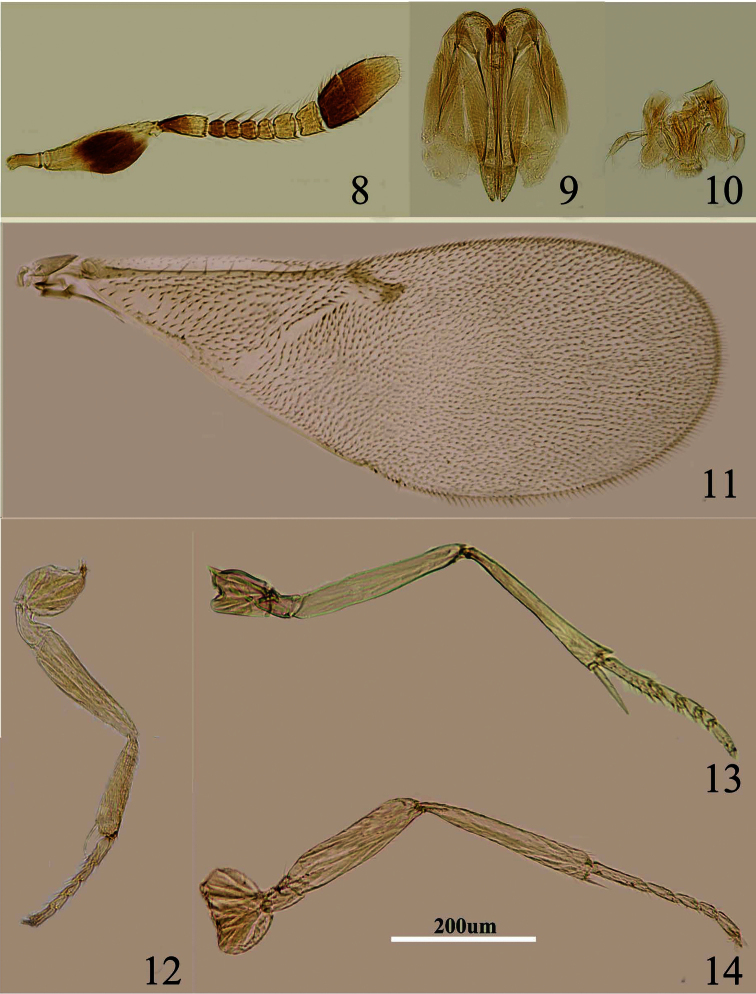
*Metaphycus alberti* (Howard) Female: **8** antenna **9** ovipositor **10** palpal formula **11** fore wing **12** fore leg **13** mid leg **14** hind leg.

Body length, including ovipositor, 0.72–1.1 mm. Frontovertex pale orange; orange in ocellar area, pale orange between occipital margin and posterior ocelli; immaculate from occiput to base of mandible; occiput with a large dark brown area above foramen, rest white; antenna (Fig. 8) with radicle very pale brown; scape mostly pale yellow and with a dark brown mark in middle, dorsal margin pale yellow; pedicel dark brown in proximal half, otherwise white, F1–F3 brown, F4–F6 white, clava dark brown, becoming slightly paler towards apex, apex pale brown; neck of pronotum brown, posterior margin translucent white, lateral spots relatively small and faint, rest white; dorsum of thorax orange; sides and posterior margin of mesoscutum and axillae inconspicuously bordered brown; setae translucent yellow, silvery in most lights; tegula white with apex pale grey-brown; metanotum orange; mesopleuron pale yellow; prosternum and mesosternum pale yellow; legs (Figs 12–14) mainly pale yellow; fore wing (Fig. 11) hyaline and with linea calva interrupted, stigmal vein about 2.3× as long as marginal vein, venation yellow-brown; hind wing hyaline; propodeum medially orange, laterally dark brown, sides white; gaster mostly yellow, sometimes pale brown dorsally from cercal plates to near apex, ovipositor sheath yellow.

Head with polygonally reticulate sculpture and mesh size slightly less than that of one eye facet; ocelli forming an acute angle less than 35°; eye not quite reaching occipital margin, separated by much less than diameter of a facet; frontovertex parallel-sided; scrobes shallow and U-shaped; antenna with scape about 2.7–3.5× as long as broad; funicle with F1–F4 smallest, subequal and transverse, F5 a little larger, F6 largest and slightly wider than long; linear sensilla only on F5 and F6; clava 3-segmented, its apex more or less rounded but with a short slightly oblique truncation; mandible relatively broad with three subequal, apical teeth; palpal formula 2-2 (Fig. 10), notaular lines reaching about 0.4× across mesoscutum; fore wing venation and setae as in Fig. 11; ovipositor (Fig. 9) slightly exserted, about 5.2× as long as ovipositor sheath.

Relative measurements: HW 13, FV 3, FVL 7, POL 2, AOL 4, OOL 1, OCL 2, POD 1, AOD 1.5, EL 8, EW 6, MS 3.5, SL 7, SW 2, FWL 37, FWW 14, HWL 26, HWW 5, OL 10, GL 2, MT 11.

Male. Length 0.7 mm. Generally similar to female but for coloration, structure of clava and genitalia. Frontovertex with ocellar area dark brown; dorsum of thorax and gaster dark brown. Antenna similar to that of female but clava solid and relatively slender; aedeagus about half as long as mid tibia.

##### Hosts.

*Coccus hesperidum* (Annecke & Mynhardt, 1981), *Coccus elongates*, *Coccus longulus* and *Ceroplastes* sp. (Hemiptera: Coccidae) (Noyes 2004).

##### Distribution.

China (Chongqing, Fujian, Guangdong, Sichuan, Zhejiang), Hawaii, USA (California), Costa Rica, South Africa, Swaziland, Australia.

##### Material examined.

China: 5♀♀, Zhejiang, Huangyan, 1964.VII.17, Coll. D. X. Liao (IZCAS); 1♀, Zhejiang, Ningbo, 2012.VII.3, F. Wang (IZCAS); 3 ♀♀, 1♂, Sichuan, Chengdu, 1961.VII.10–13, Coll. D. X. Liao (IZCAS); 2♀, Guangdong, Heyuan, 2009.XI.8, F. Yuan and Y. Z. Zhang (IZCAS); 1♀, Fujian, Wuping (Liangye Mt.), 2008.XI.15, Coll. F. Yuan (IZCAS); 1♀, Fujian, Fuzhou, 1998.X, M. Xu (IZCAS); 1♀, Chongqing, Longxi, 1992.VII.15 (BMNH). South Africa: 1♀, Zebediela, 1957–III, Coll. D. P. Annecke (BMNH).

##### Diagnosis.

Antenna with radicle very pale brown; scape mostly pale yellow and with a dark brown mark in middle, dorsal margin pale yellow; legs mainly pale yellow, scape about 2.7–3.5× as long as broad, ovipositor slightly exserted, about 5.2× as long as ovipositor sheath.*Metaphycus alberti* is very similar to *Metaphycus dispar* in general coloration and habitus.The female of *alberti* can be identified reliably by the pale yellow dorsal margin of the scape (in *dispar* dorsal margin of the scape medially interrupted by dark brown mark), and the ovipositor about 5.2× as long as ovipositor sheath (in *dispar* ovipositor about 4.3× as long as ovipositor sheath).

#### Metaphycus dispar

(Mercet, 1925)

http://species-id.net/wiki/Metaphycus_dispar

Figs 15–22

Euaphycus dispar Mercet, 1925: 25–27. Lectotype ♀ (designated by Noyes 1981: 174), Spain, IEE.
Metaphycus tamakatakaigara Tachikawa, 1957: 27–30. Holotype female, Japan, KYUN. Synonymized with *dispar* by Trjapitzin 1989: 232.
Metaphycus dispar (Mercet); Sugonjaev and Babaev 1971: 70–75. Trjapitzin 1978: 314; Trjapitzin 1989: 384; Guerrieri and Noyes 2000: 168.

##### Female.

**Figures 15–22.**
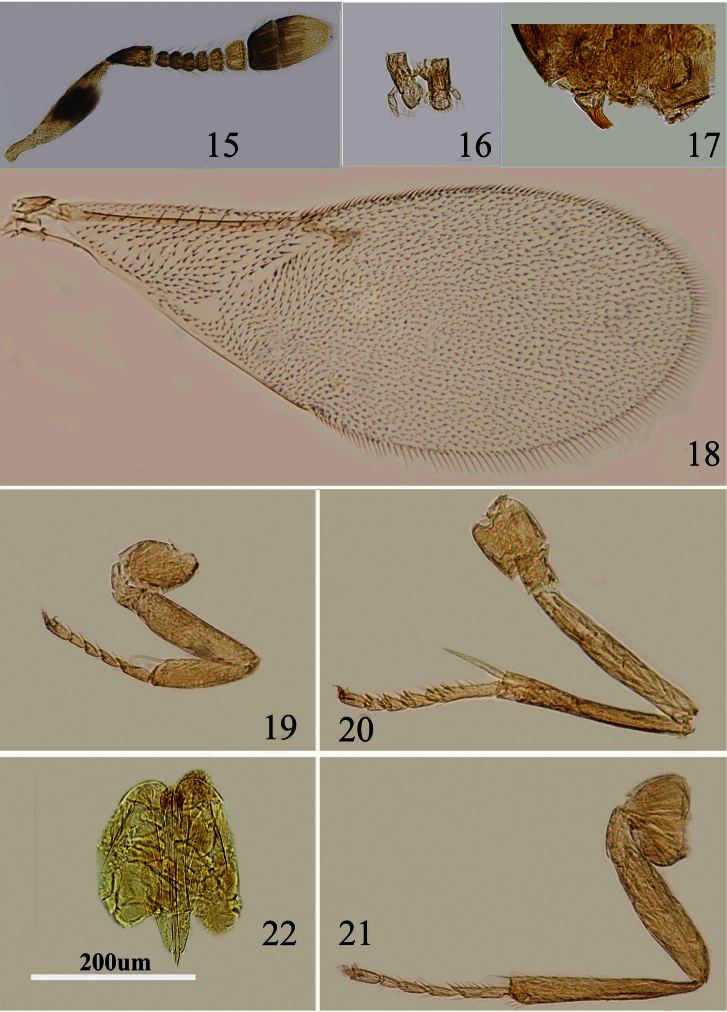
*Metaphycus dispar* (Mercet) Female: **15** antenna **16** palpal formula **17** mandible **18** fore wing **19** fore leg **20** mid leg **21** hind leg **22** ovipositor.

Body length, including ovipositor, about 0.67mm. Frontovertex orange; orange in ocellar area, pale yellow between occipital margin and posterior ocelli; immaculate from occiput to base of mandible; mouth margin pale yellow below torulus; rest of head, except occiput, white; antenna (Fig. 15) with radicle yellow; scape mostly pale yellow and with a dark brown mark in middle, dorsal margin in middle brown; pedicel in proximal two thirds dark brown and distal one third white, dark brown area extending slightly towards apex externally and internally; F1–F3 dark brown, F4 pale brown, F5–F6 yellow-white, clava dark brown, becoming slightly paler towards apex, apex yellow; occiput with a large dark brown area above foramen, rest white; neck of pronotum black, posterior margin white, lateral spots relatively large and distinct, rest white; dorsum of thorax orange; sides and posterior margin of mesoscutum and axillae inconspicuously bordered brown; setae translucent pale orange, silvery in most lights; tegula white with apex pale brown; metanotum brown; mesopleuron white; prosternum and mesosternum white; legs (Figs 19–21) pale yellow; fore wing (Fig. 18) hyaline and with linea calva interrupted, venation yellow-brown; hind wing hyaline; propodeum medially pale brown, brown laterally, sides white; gaster dorsally mainly very pale brown, but basal tergite dark brown, sides and venter white; ovipositor sheath yellow.

Head with polygonally reticulate sculpture and mesh size slightly less than that of one eye facet; ocelli forming an acute angle less than 35°; eye not quite reaching occipital margin, separated by much less than diameter of a facet; eye margins subparallel with frontovertex slightly wider anteriorly; scrobes shallow and U-shaped; antenna (Fig. 15) with scape about 3.1–3.3× as long as broad; funicle with F1–F4 smallest, subequal and transverse, F5 a little larger and F6 largest, linear sensilla only on F5 and F6; clava 3-segmented, its apex more or less rounded but with a short slightly oblique truncation; mandible relatively broad with three subequal, apical teeth (Fig. 17); palpal formula 2-2 (Fig. 16), notaular lines virtually absent; fore wing venation and setation as in Fig. 18; ovipositor (Fig. 22) slightly exserted, about 4.3× as long as ovipositor sheath.

Relative measurements: HW 9, FV 3,FVL 4, POL 2 AOL 3, OOL 0.5, OCL 1, POD 1, AOD 1, EL 6, EW 4, MS 3, SL 4.5, SW 1.5, FWL 28, FWW 11, HWL 15, HWW 4, OL 7, GL 1.6, MT 8.

##### Male.

Length 0.7–0.8 mm. Very similar to female except for antenna, genitalia and darker coloration; torulus with several pores inside the lower margin. (Guerrieri and Noyes 2000).

##### Hosts.

*Ericerus pela*, *Eulecanium* sp., *Eulecanium douglasi*, *Eulecanium kunoense*, *Eulecanium rugulosum*, *Eulecanium secretum*, *Eulecanium tiliae*, *Parthenolecanium corni*, *Parthenolecanium persicae*, *Pulvinaria* sp., *Pulvinaria vitis*, *Rhodococcus turanicus*.

##### Distribution.

China (Beijing, Xinjiang), Japan, Kazakhstan, Kirgizia, Kyrgyzstan, Mongolia, Tajikistan, Turkmenistan, Uzbekistan, Armenia, Canary Islands, Czech Republic, Finland, France, Greece, Hungary, Italy, Madeira, Russia, Russia-Adygeyskaya, Russia-Altayskiy Kray, Russia-Buryatskaya Respublika, Russia-Sakhalin Oblast, Slovakia, Spain, United Kingdom, United Kingdom-England, former Yugoslavia, Georgia, USA, (Trjapitzin 1989; Guerrieri and Noyes 2000).

##### Material examined.

China: 1♀, Xinjiang, Kurle, 1965.VI.9, Coll. D. X. Liao (IZCAS); 1♀, Beijing, Changping, 2011.IX.23 (IZCAS); 1♀, Jiangsu, Nianjing, 2011.VI. (IZCAS); 1♀, Liaoning, Shenyang, 1991.VI., Coll. J. X. Lou (IZCAS). France: 1♀, Corsica Propriano, 1989.VIII, Coll. J. S. Noyes (BMNH); Greece: 1♀, Corfu Ano Kourakiana, 1987.VIII.30, Coll. J. S. Noyes (BMNH); Japan: 1♀, Tokyo, 1981.VIII.2, Coll. Ikece & Carlson (BMNH).

##### Diagnosis.

Scape mostly pale yellow and with a dark brown mark in middle, dorsal margin in middle brown, and about 3.1–3.3× as long as broad; ovipositor slightly exserted, about 4.3× as long as ovipositor sheath.See diagnosis under the *Metaphycus alberti*. According to Guerrieri and Noyes (2000), *Metaphycus dispar* is very close to *Metaphycus kozari* Sugonjaev (1975).

#### Metaphycus chinensis sp. n.

urn:lsid:zoobank.org:act:0D6FA99A-77F0-4525-83B7-AA1B93277977

http://species-id.net/wiki/Metaphycus_chinensis

Figs 23–29

##### Holotype.

♀, China, Jiangsu, Nanjing: 2011.VI.1, coll. L. Ding (IZCAS).

##### Paratypes.

2♀♀, the same as holotype (IZCAS).

**Figures 23–29.**
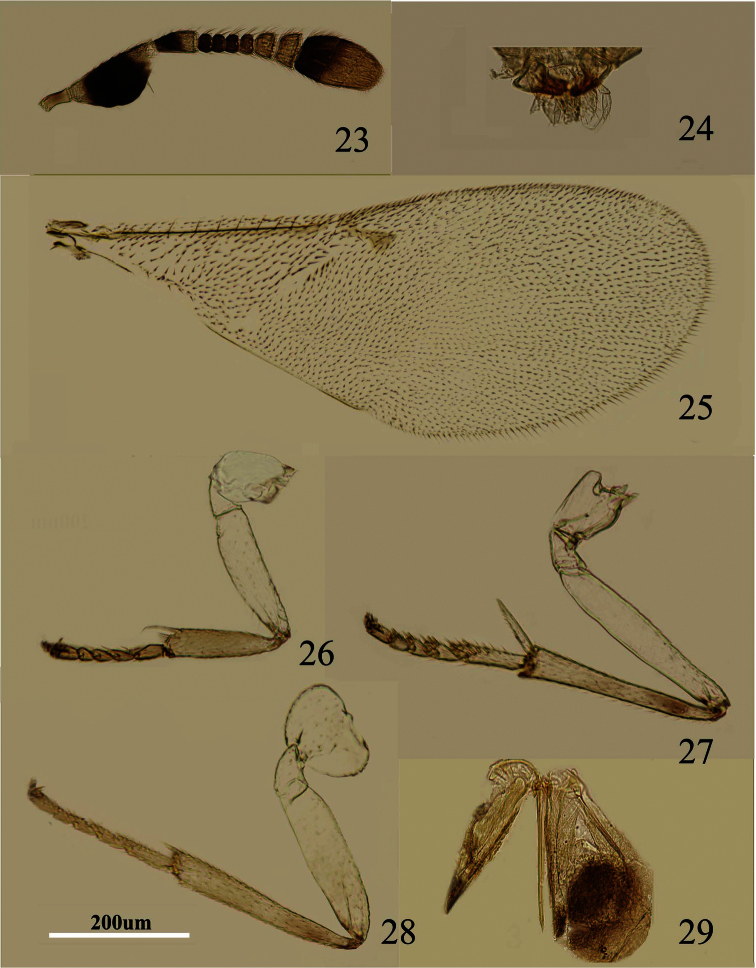
*Metaphycus chinensis* sp. n. Female: **23** antenna **24** palpal formula **25** fore wing **26** fore leg **27** mid leg **28** hind leg **29** ovipositor.

**Female:** Body length, including ovipositor, 0.7mm. Frontovertex orange; orange in ocellar area, yellow between occipital margin and posterior ocelli; immaculate from occiput to base of mandible; rest of head, except occiput, white; antenna (Fig. 23) with radicle brown; scape with both faces blackish, extreme base and apex yellow; pedicel dark brown in proximal half, otherwise white; F1–F3 dark brown, F4 pale brown, F5–F6 white, clava dark brown, becoming paler towards apex, apex paler brown; occiput with a large dark brown area above foramen, rest white; neck of pronotum black, posterior margin translucent brown, lateral spots relatively large and distinct, rest white; dorsum of thorax orange; sides and posterior margin of mesoscutum and axillae inconspicuously bordered pale brown; setae translucent orange, silvery in most lights; tegula white with apex pale grey-brown; metanotum pale brown; mesopleuron pale yellow; prosternum and mesosternum white; legs (Figs 26–28) mainly pale yellow, occasionally mid and hind tibiae with faint brown marking; fore wing (Fig. 25) hyaline and with linea calva interrupted, venation yellow-brown; hind wing hyaline; propodeum medially dark brown, laterally white; dorsum of gaster brown but T8 white, sides and venter white; ovipositor sheath yellow.

Head with polygonally reticulate sculpture and mesh size slightly less than that of one eye facet; ocelli forming an angle of about 40°; eye not quite reaching occipital margin, separated by much less than diameter of a facet; frontovertex subparallel-sided; scrobes shallow and U-shaped; lateral antennal groove absent; antenna (Fig. 23) with scape about 2.3× as long as broad; funicle with F1–F4 smallest, subequal and transverse, F5 larger but transverse, F6 largest, linear sensilla only on F5 and F6; clava 3-segmented, its apex more or less rounded but with a short slightly oblique truncation; mandible relatively broad with three subequal, apical teeth; palpal formula 2-2 (Fig. 24), notaular lines virtually absent; fore wing venation and setation as in Fig. 25; ovipositor (Fig. 29) hardly exserted, about 4.8× as long as ovipositor sheath.

Relative measurements: HW 11, FV 3, FVL 6, POL 1.5, AOL 3,OOL 0.5, OCL 1,POD 1, AOD 1, EL 7, EW 5, MS 3.5, SL 5, SW 2.2, FWL 30, FWW 11, HWL 19, HWW 3, OL 9, GL 1.9, MT 11.

**Male.** Unknown.

##### Host.

Unknown**.**

##### Distribution.

China (Jiangsu).

##### Etymology.

The specific epither of this new species is derived from the type locality “China”.

##### Diagnosis.

Antenna with radicle brown; scape with both faces blackish, extreme base and apex yellow; scape about 2.3× as long as broad; dorsum of gaster brown but T8 white, sides and venter white; ovipositor sheath yellow; ovipositor hardly exserted, about 4.8× as long as ovipositor sheath.This species is close to *Metaphycus ericeri* in appearance. It can be separated from the latter as follows: fore wing 2.7× as long as broad (Fig. 25), ocelli forming an angle of about 40°; ovipositor (Fig. 29) about 4.8× as long as ovipositor sheath (in *ericeri*, fore wing 2.4× as long as broad (Fig. 53), ocelli forming an angle of about 50°; ovipositor (Fig. 57) about 5.4× as long as ovipositor sheath).

#### Metaphycus wui sp. n.

urn:lsid:zoobank.org:act:9C8CB15E-29C7-40C1-8CAD-DA6EBAEA2AE6

http://species-id.net/wiki/Metaphycus_wui

Figs 30–38

##### Holotype.

♀, China, Guangxi, Chongzuo, 2011.IV.22, ex. *Ceroplastes* sp., coll. S. A. Wu (IZCAS).

##### Paratypes.

2♀♀, same as holotype (IZCAS).

**Figures 30–38.**
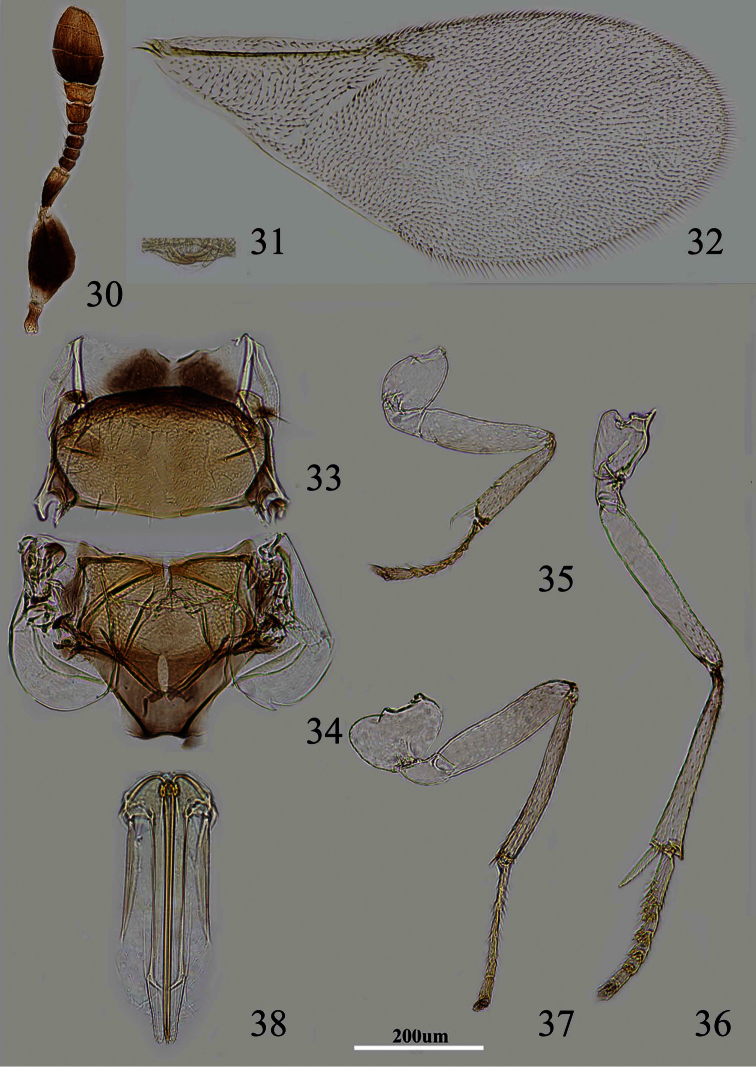
*Metaphycus wui* sp. n. Female: **30** antenna **31** palpal formula **32** fore wing **33** mesoscutum **34** scutellum **35** fore leg **36** mid leg **37** hind leg **38** ovipositor.

**Female:** Body length, including ovipositor, 0.7–0.8mm. Frontovertex orange; orange in ocellar area, orange between occipital margin and posterior ocelli; gena with brown-yellow from occiput to base of mandible; mouth margin yellow below torulus; rest of head, except occiput, white; antenna (Fig. 30) with radicle very pale brown; scape mostly black only extreme base and apex white; pedicel dark brown in proximal half otherwise white, F1–F4 dark brown, F5–F6 white, clava dark brown, becoming slightly paler towards apex, apex paler brown; occiput with a large black area above foramen, rest white;neck of pronotum black, posterior margin brown, lateral spots relatively large and distinct, rest brown; dorsum of thorax brown-yellow; sides and posterior margin of mesoscutum and axillae inconspicuously bordered yellow-brown (Fig. 34); setae translucent yellow, silvery in most lights;tegula white only apex pale brown; metanotum dark brown; mesopleuron pale yellow; prosternum and mesosternum white; legs (Figs 35–37) mainly pale brown-yellow; fore wing (Fig. 32) hyaline, venation yellow-brown; hind wing hyaline and with linea calva interrupted; propodeum medially dark brown; gaster dorsally brown, side and venter white; ovipositor sheath pale yellow.

Head with polygonally reticulate sculpture and mesh size slightly less than that of one eye facet; ocelli forming an angle of about 45°; eye not quite reaching occipital margin, separated by much less than diameter of a facet; eye margins subparallel with frontovertex slightly wider anteriorly; scrobes shallow and U-shaped; antenna (Fig. 30) with scape about 2.3× as long as broad; funicle with F1–F4 smallest, subequal and transverse, F5 a little larger, F6 largest and quadrate, linear sensilla only on F5 and F6; clava 3-segmented, its apex rounded; mandible relatively broad with three subequal, apical teeth; palpal formula 2-2 (Fig. 31), notaular lines reaching about 0.5× across mesoscutum (Fig. 33); fore wing venation and setation as in Fig. 32; ovipositor (Fig. 38) slightly exserted, about 4.7× as long as ovipositor sheath.

Relative measurements: HW 12, FV 3,FVL 6, POL 2, AOL 3, OOL 1, OCL 1,POD 1, AOD 1, EL 8, EW 5,MS 2, SL 7, SW 3, FWL 36, FWW 17, OL 16, GL 3.4, MT 12.

**Male.** Unknown.

##### Host.

*Ceroplastes* sp. on *Pinus*.

##### Distribution.

China (Guangxi).

##### Etymology.

The species is named after Professor Sanan Wu, who helped to identify many hosts of Encyrtidae.

##### Diagnosis.

Antenna with radicle very pale brown; scape mostly black, only extreme base and apex white, about 2.3× as long as broad; legs mainly pale brown-yellow; ovipositor slightly exserted, about 4.7× as long as ovipositor sheath.This species is similar to *Metaphycus fusiscapus* in colour and size. It can be separated from the latter as follows: scape about 2.3× as long as broad (in *fusiscapus*, scape about 2× as long as broad); mid and hind tibiae immaculate (Fig. 36), at most with a fuscus spot near base of mid tibiae (in *fusiscapus* mid and hind tibiae with distinct dark brown marking).

#### Metaphycus stylatus sp. n.

urn:lsid:zoobank.org:act:B8D8DF2C-283E-447F-9776-E361F4A669C0

http://species-id.net/wiki/Metaphycus_stylatus

Figs 39–47

##### Holotype.

♀, China, Beijing: 2011.VII.1 (IZCAS).

##### Paratypes.

2♀♀, same as holotype (IZCAS).

**Figures 39–47.**
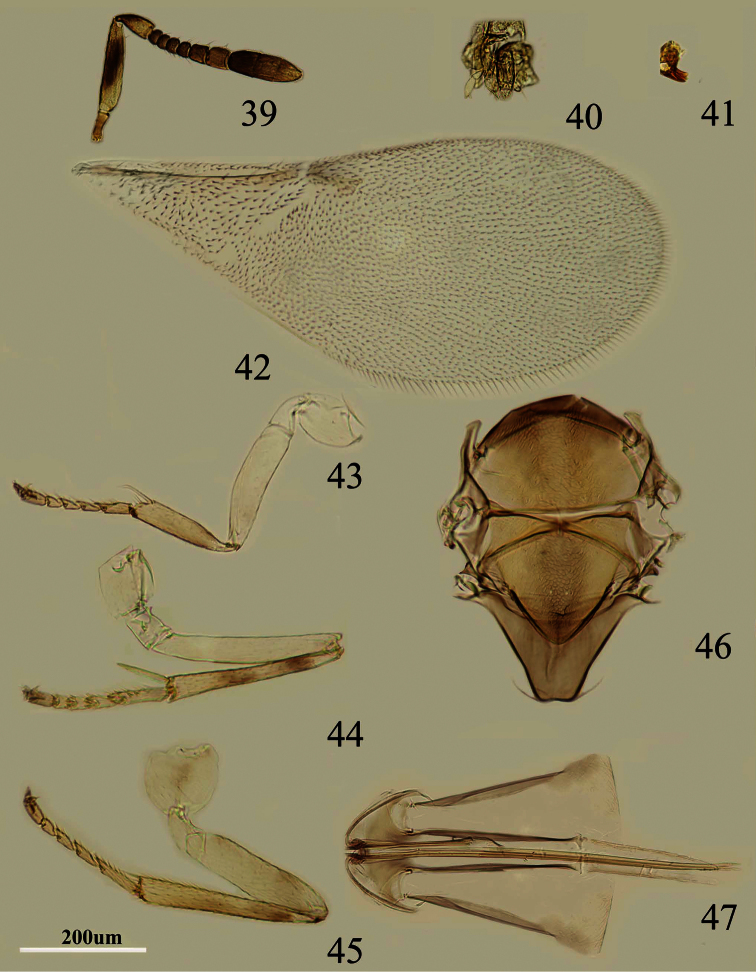
*Metaphycus stylatus* sp. n. Female: **39** antenna **40** palpal formula **41** mandible **42** fore wing **43** fore leg **44** mid leg **45** hind leg **46** mesothorax **47** ovipositor.

**Female:** Body length, including ovipositor about 0.8mm. Frontovertex orange; orange in ocellar area, orange between occipital margin and posterior ocelli; gena with a fairly broad, oblique, pale brown-yellow from occiput to base of mandible; mouth margin narrowly pale brown below torulus; rest of head, except occiput, white; antenna (Fig. 39) with radicle yellow; scape mostly dark brown and base white; pedicel dark brown in proximal half, otherwise white, F1–F4 dark brown, F5–F6 pale brown-grey, clava dark brown, becoming slightly paler towards apex, apex paler brown; occiput with a large dark brown area above foramen, rest white; neck of pronotum pale black, posterior margin brown, lateral spots relatively large and distinct, rest white; dorsum of thorax yellow-brown; sides and posterior margin of mesoscutum and axillae inconspicuously bordered brown; setae translucent yellow, silvery in most lights; tegula white and apex grey-brown; metanotum dark brown; mesopleuron pale yellow; prosternum and mesosternum white; legs (Figs 43–45) mainly pale yellow but tibiae at knees narrowly dark brown, mid and hind tibiae with a pair of dark brown rings at about 0.2× and 0.5×; fore wing (Fig. 42) hyaline and with linea calva interrupted, venation yellow-brown; hind wing hyaline; propodeum medially brown, laterally yellow; gaster dorsally brown, sides and venter white; ovipositor sheath yellow.

Head with polygonally reticulate sculpture and mesh size slightly less than that of one eye facet; ocelli forming an angle of about 60°; eye not quite reaching occipital margin, separated by one diameter of a facet; eye margins subparallel with frontovertex slightly wider anteriorly; scrobes shallow and U-shaped; antenna with scape about 5.5× as long as broad; funicle with F1–F4 smallest, subequal and transverse, F5 and F6 largest, subequal; linear sensilla only on F5 and F6; clava 3-segmented, its apex rounded; mandible relatively broad with three subequal, apical teeth (Fig. 41); palpal formula 2-2 (Fig. 40), notaular lines reaching about 0.6× across mesoscutum (Fig. 46); fore wing venation and setation as in Fig. 42; ovipositor (Fig. 47) strongly exserted, the exserted part about 2.6× as long as mid tibial spur, ovipositor length about 2.8× as long as ovipositor sheath.

Relative measurements: HW 11, FV 4,FVL 5, POL 2,AOL 2, OOL 1, OCL 1, POD 1, AOD 1, EL 7, EW 4, MS 3, SL 5.5, SW 1, FWL 31, FWW 12, HWL 20, HWW 3.5, OL 15, GL 5.4, MT 9.

**Male.** Unknown.

**Host.** Unknown.

##### Distribution.

China (Beijing).

##### Etymology.

The specific epithet is derived from the latin word “stylatus” referring to the long ovipositor sheath of the new species.

##### Diagnosis.

Scape mostly dark brown and base white and about 5.5× as long as broad; legs mainly pale yellow but tibiae at knees narrowly dark brown, mid and hind tibiae with a pair of dark brown rings at about 0.2× and 0.5×; gaster dorsally brown, sides and venter white; ovipositor sheath strongly exserted, the exserted part about two thirds gaster length; ovipositor length about 2.8× as long as ovipositor sheath.

*Metaphycus stylatus* differs from other species studied here by the strongly exserted ovipositor sheath, which is about two thirds gaster length. In other species, the ovipositor sheath is less than one fifth the gaster length. Using the key of Guerrieri and Noyes (2000), this species runs to *Metaphycus asterolecanii* (key couplet 12). It can be separated from *asterolecanii* as follows: scape about 5.5× as long as broad; head about 3× as broad as frontovertex, and ocelli forming an angle of about 60°; ovipositor (Fig. 47) strongly exserted, about 1.7× as long as mid tibia (in *asterolecanii*, scape about 3× as long as broad; head about 4× as broad as frontovertex, and ocelli forming an angle of clearly less than 60°; ovipositor hidden and nearly as long a mid tibia).

#### Metaphycus ericeri

Trjapitzin, 1967

http://species-id.net/wiki/Metaphycus_ericeri

Figs 48–57

Metaphycus ericeri Trjapitzin, 1967: 185. Holotype ♀, Russia–Primor‘ye Kray.
Metaphycus ericeri Trjapitzin: Trjapitzin 1975: 10; Trjapitzin 1989: 345.

##### Female.

**Figures 48–57.**
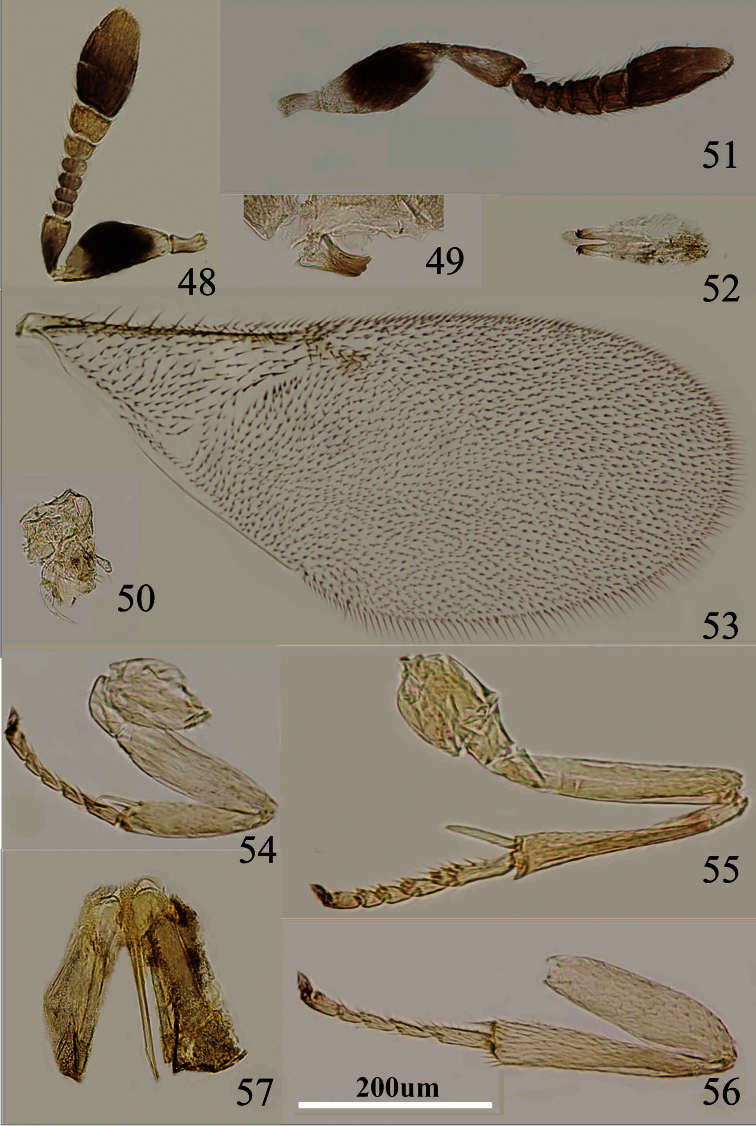
*Metaphycus ericeri* Trjapitzin Female: **48** antenna **49** mandible **50** palpal formula **53** fore wing **54** fore leg **55** mid leg **56** hind leg **57** ovipositor. Male: **51** antenna **52** genitalia.

Body length, including ovipositor about 0.8 mm. Frontovertex pale orange; pale brown in ocellar area, yellow between occipital margin and posterior ocelli; immaculate from occiput to base of mandible; rest of head, except occiput, white; antenna (Fig. 48) with radicle brown-yellow; scape with both faces blackish, only extreme apex and extreme distal yellow, dorsal margin pale black; pedicel dark brown in proximal half, otherwise white; F1–F4 dark brown, F5–F6 white, clava dark brown, becoming paler towards apex, apex paler brown; occiput with a large dark brown area above foramen, rest white; neck of pronotum dark brown, posterior margin translucent white, lateral spots relatively large and distinct, rest white; dorsum of thorax orange; sides and posterior margin of mesoscutum and axillae inconspicuously bordered pale brown; setae translucent orange, silvery in most lights; tegula white with apex pale grey-brown; metanotum orange; mesopleuron pale yellow; prosternum and mesosternum pale yellow; legs (Figs 54–56) mainly pale yellow and mid tibia with a faint brown marking; fore wing (Fig. 53) hyaline and with linea calva interrupted, venation yellow-brown; hind wing hyaline; propodeum medially orange; gaster mostly orange but brown dorsally from cercal plates to near apex, ovipositor sheath yellow.

Head with polygonally reticulate sculpture and mesh size slightly less than that of one eye facet; ocelli forming an angle of about 50°; eye not quite reaching occipital margin, separated by much less than diameter of a facet; frontovertex not parallel-sided, becoming slightly broader anteriorly from the narrowest point which is slightly in front of posterior ocelli; scrobes shallow and U-shaped; antenna with scape about 2.3× as long as broad; funicle with F1–F4 smallest, subequal and transverse, F5 a little larger but transverse, F6 largest, linear sensilla only on F5 and F6; clava 3-segmented, its apex more or less rounded but with a short slightly oblique truncation; mandible relatively broad with three subequal, apical teeth (Fig. 49); palpal formula 2-2 (Fig. 50), thorax dorsally with notaular lines present only anteriorly; fore wing venation and setation as in Fig. 53; ovipositor (Fig. 57) hardly exserted, about 5.4× as long as ovipositor sheath.

Relative measurements: HW 14, FV 4, FVL 9,POL 2, AOL 3, OOL 1, OCL 1, POD 1, AOD 1, EL 8, EW 6, MS 5, SL 7, SW 3, FWL 36, FWW 15, HWL 24, HWW 5, OL 8, GL 1.5, MT 13.

**Male.** Length 0.65–0.70mm, almost identical to female but for genitalia and antenna, coloration of gaster. Pedicel white and with clava relatively more slender and solid, funicle brown (Fig. 51); genitalia (Fig. 52) with digitus long and slender apically with two hooks; aedeagus sharply pointed at apex.

##### Distribution.

China (Liaoning), Russia.

##### Host.

*Ericerus pela*.

##### Material examined.

China: 3 ♀♀, 6 ♂♂, Liaoning, Xiuyan, 2010.VI, Coll. Y. Q. Xi (IZCAS); 2 ♀♀, 1 ♂, Liaoning, Shenyang, 2009.VI.12, Coll. Y. Q. Xi (IZCAS).

##### Diagnosis.

Scape about 2.3× as long as broad; legs mainly pale yellow and mid tibia with a faint brown marking; gaster mostly orange but brown dorsally from cercal plates to near apex, ovipositor sheath yellow; ovipositor about 5.4× as long as ovipositor sheath.

This species is similar to *Metaphycus helvolus* in appearance. It can be separated from *Metaphycus helvolus* as follows: dorsal margin of scape pale black; linear sensilla on F5 and F6 (in *helvolus* dorsal margin of scape yellowish, linear sensilla absent on F5), head is about 3.5× as broad as frontovertex, the ovipositor about 0.6× as long as mid tibia (in *helvolus* head about 3× as broad as frontovertex, and the ovipositor about as long as mid tibia).

#### Metaphycus nadius

(Walker, 1838)

http://species-id.net/wiki/Metaphycus_nadius

Figs 58–62

Encyrtus nadius Walker, 1838: 423. Lectotype ♀ (BMNH, examined), designated by Bouček and Graham (1978: 230), England.
Encyrtus syllaeus Walker, 1838b: 426. Lectotype ♂ (designated by Bouček and Graham 1978: 230), England, BMNH, examined. Synonymized by Guerrieri and Noyes 2000: 158.
Aphycus pinicola Mercet, 1917: 135. Lectotype ♀ (designated by Noyes 1981: 168), Spain, IEEM. Synonymized with *nadius* by Guerrieri and Noyes 2000: 158.
Aphycus (Euaphycus) pinicola Mercet; Mercet 1921: 205.
Euaphycus intermedius Mercet, 1925: 24. Synonymized with *nadius* by Guerrieri and Noyes 2000: 158.
Euaphycus callunae Alam, 1957: 433. Holotype ♀, England, BMNH. Synonymized with *nadius* by Guerrieri and Noyes 2000: 158.
Euaphycus duplus Chumakova, 1961: 324. Synonymized with *nadius* by Guerrieri and Noyes 2000: 158.
Metaphycus intermedius (Mercet): Trjapitzin 1975: 8.
Metaphycus callunae (Alam); Trjapitzin 1975: 13.
Metaphycus pinicola (Mercet); Trjapitzin 1975: 14.
Metaphycus duplus (Chumakova); Trjapitzin 1975: 14.
Metaphycus nadius ; Bouček and Graham 1978: 230; Trjapitzin 1989: 246; Li and Xu 2006: 112–113.
Metaphycus syllaeus (Walker); Bouček and Graham 1978: 230.

##### Female.

**Figures 58–62.**
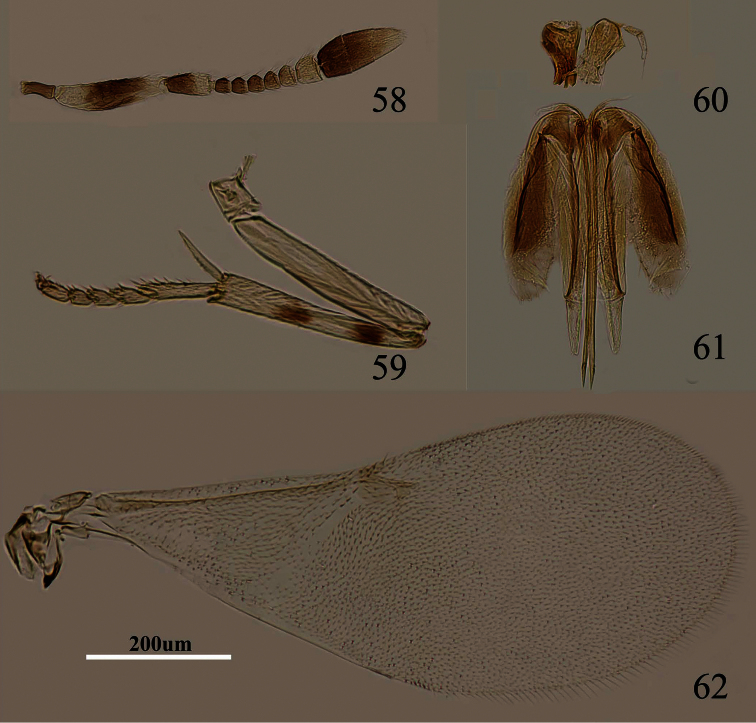
*Metaphycus nadius* (Walker) Female: **58** antenna **59** mid leg **60** palpal formula **61** ovipositor **62** fore wing.

Body length, including ovipositor, 0.7–0.8mm. Frontovertex dark orange; brown in ocellar area, brown between occipital margin and posterior ocelli; dark brown from occiput to base of mandible; mouth margin narrowly dark brown below torulus; rest of head, except occiput, brown; antenna (Fig. 58) with radicle dark brown; scape with both faces dark brown and base of scape white; pedicel in proximal half dark brown, distal half white, dark brown area extending slightly towards apex externally and internally; F1–F4 pale brown, F5–F6 white, clava dark brown, becoming slightly paler towards apex, apex paler brown; occiput with a large dark brown area above foramen; neck of pronotum black, posterior margin translucent brown; dorsum of thorax dark brown; sides and posterior margin of mesoscutum and axillae inconspicuously bordered brown; setae translucent pale brown, silvery in most lights; tegula pale brown with apex pale darker; metanotum dark brown; mesopleuron pale brown; prosternum and mesosternum brown; legs (Fig. 59) mainly pale yellow but tibiae at knees narrowly dark brown and each with a pair of dark brown rings at about 0.2× and 0.5× (fore tibia at about 0.5×); fore wing (Fig. 62) hyaline with a small infuscate area beneath stigmal vein, and with linea calva interrupted; venation yellow-brown; hind wing hyaline; propodeum dark brown; gaster dorsally and venter dark brown, sides very pale brown to white; ovipositor sheath pale brown.

Head with polygonally reticulate sculpture and mesh size slightly less than that of one eye facet; ocelli forming an angle of about 45°; eye not quite reaching occipital margin, separated by much less than diameter of a facet; eye margins subparallel; scrobes shallow and U-shaped; antenna with scape about 4.5× as long as broad; funicle with F1–F4 smallest, subequal and transverse, F5 a little larger but transverse, F6 largest and quadrate; linear sensilla only on F6; clava 3-segmented, its apex more or less rounded but with a short slightly oblique truncation; mandible relatively broad, with three subequal, apical teeth; palpal formula 2-2 (Fig. 60), gaster with ovipositor slightly exserted, notaular lines reaching about 0.7× across mesoscutum; fore wing venation and setation as in Fig. 62; ovipositor (Fig. 61) clearly exserted, about 4.3× as long as ovipositor sheath.

Relative measurements: HW 12, FV 3, FVL 4, POL 1.5, AOL 2, OOL 1, OCL 0.5, POD 1, AOD 1, EL 9, EW 5, MS 3, SL 6, SW 2, FWL 32, FWW 15, HWL 22, HWW 4, OL 11, GL 2.5, MT 10.

**Male.** Almost identical to female in general structure, habitus and coloration except for solid clava, genitalia.

##### Hosts.

*Asterolecanium* sp.; *Asterolecanium minus*; *Chionaspis pinifoliae*; *Diaspidiotus bavaricus*; *Metaphycus gigas*; *Metaphycus zonatus*; *Phenacaspis pinifoliae*; *Quadraspidiotus bavaricus*; *Quadraspidiotus gigas*; *Quadraspidiotus perniciosus*; *Quadraspidiotus zonatus*; *Sphaerolecanium prunastri*.

##### Distribution.

China (Heilongjiang, Inner Mongolia, Qinghai); Croatia, Czech Republic, England, Finland, France, Greece, Hungary, Italy, Netherlands, Poland, Portugal, Wales, Russia, Slovakia, Spain, United Kingdom.

##### Material examined.

China: 1♀, Inner Mongolia: Darhan Maomingan Allied county, 1979.VIII.1 (IZCAS); 7♀♀, 13♂♂, Qinghai Geermu, 2007.VII.4–5 (IZCAS), England: 1♀, 1985, Coll. S. M. Alam (BMNH); 1♀, Richmond Park, Surrey, 1996.VII.18, Coll. J. Noyes (BMNH).

##### Diagnosis.

Antenna with radicle dark brown; scape with both faces dark brown and base of scape white; scape about 4.5× as long as broad; legs mainly pale yellow but tibiae at knees narrowly dark brown and each with a pair of dark brown rings at about 0.2× and 0.5× (fore tibia at about 0.5×); fore wing hyaline with a small infuscate area beneath stigmal vein.The female of *Metaphycus nadius* can be identified reliably from other Chinese species in this group by the brown mark under the stigmal vein and the two rings on the mid tibia, antenna with linear sensilla on F6 and clava only. According to Guerrieri and Noyes (2000), it is also similar to *Metaphycus hubai*, both with a small infuscate area below marginal and stigma veins.

#### Metaphycus fusiscapus sp. n.

urn:lsid:zoobank.org:act:A309BC1D-C062-460D-B0CA-5BDB716D77F2

http://species-id.net/wiki/Metaphycus_fusiscapus

Figs 63–69

##### Holotype.

♀, China, Sichuan, Chengdu: 2012.VI.30, ex. *Ceroplastes floridensis*, coll. J. Deng (IZCAS).

##### Paratypes.

3♀♀, same as holotype. 1 ♀, Fujian, Shaowu, 2012.IV.17, ex. *Ceroplastes floridensis*, coll. A. K. Deng (IZCAS).

**Figures 63–69.**
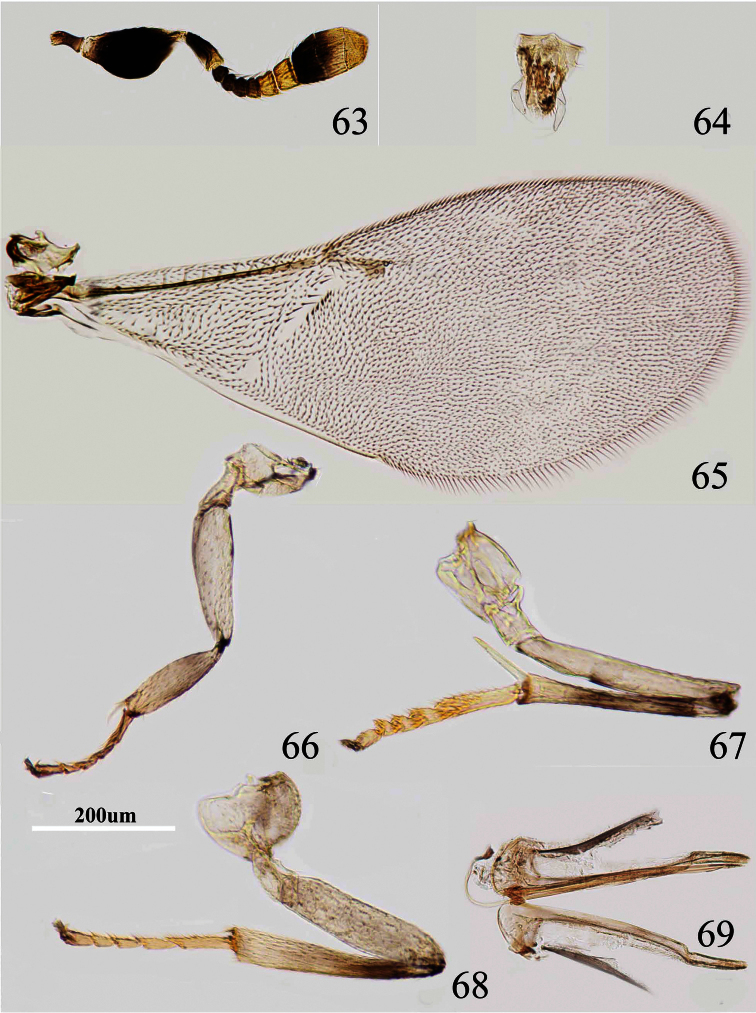
*Metaphycus fusiscapus* sp. n. Female: **63** antenna **64** palpal formula **65** fore wing **66** fore leg **67** mid leg **68** hind leg **69** ovipositor.

**Female:** Body length, including ovipositor, 0.9–1mm. Frontovertex orange; very pale brown in ocellar area, pale brown between occipital margin and posterior ocelli; gena with brown-grey; mouth margin narrowly pale brown below torulus; rest of head, except occiput, white; antenna (Fig. 63) with radicle dark brown; scape with both faces black, dorsal margin black, extreme apex white; pedicel in proximal four fifths dark brown, distal one fifth white, dark brown area extending slightly towards apex externally and internally; F1–F3 dark brown, F4 pale brown to pale yellow, F5–F6 white, clava proximal half dark brown, becoming white towards apex; neck of pronotum dark brown, posterior margin translucent brown, lateral spots relatively large and distinct, rest white; dorsum of thorax dark orange; sides and posterior margin of mesoscutum and axillae inconspicuously bordered brown; scutellum slightly darker in center; setae translucent pale brown, silvery in most lights; tegula white with apex brown; metanotum black; mesopleuron white; prosternum and mesosternum pale brown; legs (Figs 66–68) with insides white, and outsides very pale brown, coxae white, but tibiae at knees narrowly dark brown and fore tibia with faint brown rings, mid and hind tibiae with a pair of dark brown rings at about 0.2× and 0.5×; fore wing (Fig. 65) hyaline and venation brown; hind wing hyaline, and with linea calva interrupted; propodeum medially black, laterally white; gaster dorsally black, sides and venter white; ovipositor sheath pale brown.

Head ocelli forming an angle of about 40°; eye not quite reaching occipital margin, separated by much less than diameter of a facet; frontovertex parallel-sided; scrobes shallow and U-shaped; antenna (Fig. 63) with scape about 2× as long as broad; funicle with F1–F4 smallest, subequal and transverse, F5 a little larger, F6 largest and quadrate; linear sensilla only on F5 and F6; clava 3-segmented, its apex more or less rounded but with a short slightly oblique truncation; mandible relatively broad with three subequal, apical teeth; palpal formula 2-2 (Fig. 64). notaular lines reaching about 0.5× across mesoscutum; fore wing venation and setation as in Fig. 65; ovipositor (Fig. 69) hardly exserted, about 4.1× as long as ovipositor sheath.

Relative measurements: HW 11, FV 4, FVL 6, POL 2, AOL 2.5, OOL 1, OCL 1, POD 1, AOD 1, EL 7, EW 5, MS 3, SL 6, SW 3, FWL 40, FWW 15**,** HWL 22, HWW 6, OL 14, GL 3.5, MT 12.

**Male.** Unknown.

##### Host.

*Ceroplastes floridensis*.

##### Distribution

**.** China (Sichuan, Fujian).

##### Etymology.

This specific epither of this new species is referring to the dark brown scape.

##### Diagnosis.

Scape with both faces black, dorsal margin black, extreme apex white, and about 2× as long as broad; legs (Figs 66–68) with inner sides white, and outer sides very pale brown, coxae white, but tibiae at knees narrowly dark brown and fore tibia with faint brown rings, mid and hind tibiae with a pair of dark brown rings at about 0.2× and 0.5×; ovipositor hardly exserted, about 4.1× as long as ovipositor sheath.

Using the key of Guerrieri and Noyes (2000), *Metaphycus fusiscapus* runs to *Metaphycus pretiosus* (key couplet 8), but can be separated from the latter by scape about 2× as long as broad and head 4.1× as broad as frontovertx (in *pretiosus*, scape about 4× as long as broad, head 3× as broad as frontovertex). Using the key of Zeya and Hayat (1993), this species goes to *Metaphycus agarwali*. Both of these two species having two dark bands on mid tibiae and scape about 2× as long as broad. It can be separated from *Metaphycus agarwali* as follows: dorsal margin of scape black only base apex white, F1–F4 subequal and F5 distinctly larger (in *Metaphycus agarwali*, scape dorsal margin white, and F1–F5 subequal). Both of the two species are distributed in Asia, perhaps they are closely related. In China, *Metaphycus fusiscapus* is very similar to *Metaphycus wui* (see comments under *Metaphycus wui*).

#### Metaphycus fusiformis sp. n.

urn:lsid:zoobank.org:act:7199B2CC-9C8B-4DB7-8680-CE252E87C303

http://species-id.net/wiki/Metaphycus_fusiformis

Figs 70–76

##### Holotype.

1♀, China, Shanxi, Li Mt.: 2006.VIII.1, Coll. D. Liu (IZCAS).

##### Paratypes.

1♀, Beijing, Mentougou, 2011.VIII.30 (IZCAS); 2♀♀, Beijing, Changping, 2009.VIII.7, Coll. F. Yuan (IZCAS), 2♀♀, Beijing, Changping, 2009.VIII.7, Coll. Q. T. Wu (IZCAS), 1♀, Hainan, Danzhou, 2005.I, Coll. T. X Zhang (IZCAS); 1♀, Hainan, Danzhou, 2007.V.16, Coll. Y. Z. Zhang (IZCAS).

**Figures 70–76.**
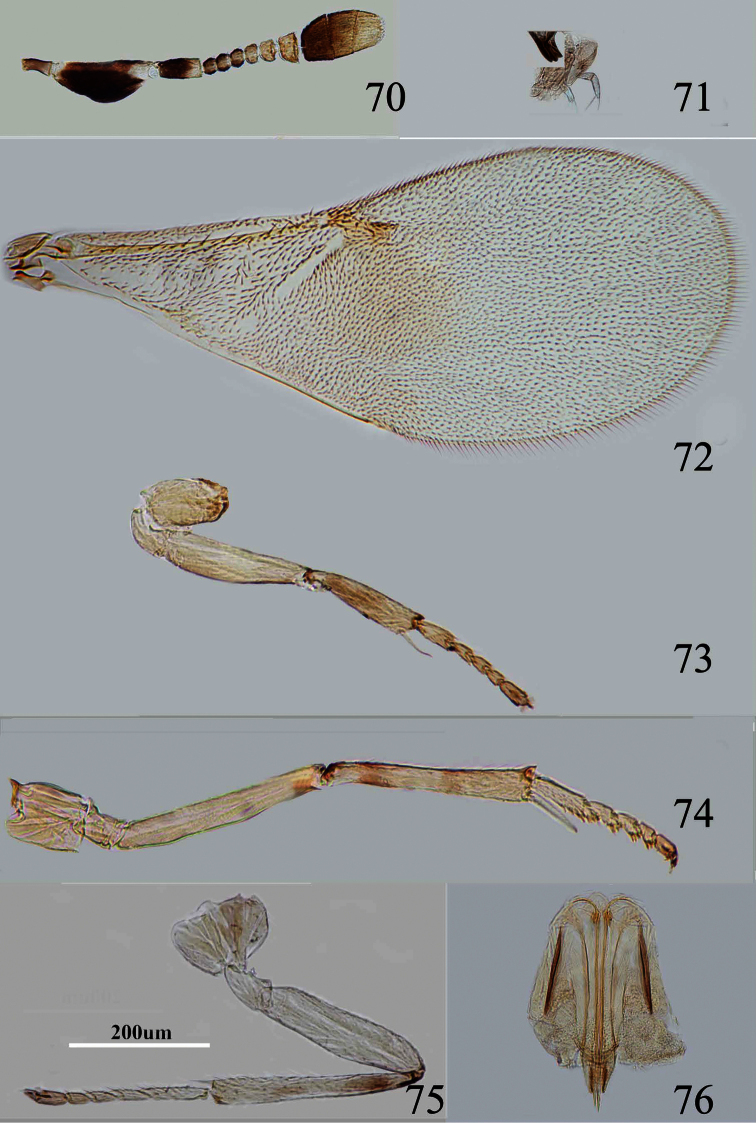
*Metaphycus fusiformis* sp. n. Female: **70** antenna **71** palpal formula **72** fore wing **73** fore leg **74** mid leg **75** hind leg **76** ovipositor.

**Female:** Body length, including ovipositor about 0.8–0.9mm. Frontovertex orange; orange in ocellar area, very pale brown to orange between occipital margin and posterior ocelli; gena with a fairly broad, oblique**,** brown mark from occiput to base of mandible; mouth margin narrowly pale brown below torulus; rest of head, except occiput, white; antenna (Fig. 70) with radicle dark brown; scape with both faces dark brown, blackish, dorsal margin narrowly pale yellow, extreme apex white; pedicel base at most two thirds dark brown, white distally, dark brown area extending slightly towards apex externally and internally; F1–F3 dark brown, F4 brown, F5–F6 white-yellow, clava in proximal 2/3 dark brown, becoming slightly paler towards apex, apex yellow; occiput with a large dark brown area above foramen, rest pale yellow; neck of pronotum dark brown, posterior margin translucent yellow, lateral spots relatively large and distinct, rest orange; dorsum of thorax orange; sides and posterior margin of mesoscutum and axillae conspicuously bordered brown; setae translucent pale brown, silvery in most lights; tegula white with apex pale grey-brown; metanotum brown; mesopleuron pale yellow; prosternum yellow and mesosternum white; legs (Fig. 73–75) mainly white but tibiae at knees narrowly dark brown and mid and hind tibiae with a pair of dark brown rings at about 0.2× and 0.5×; fore wing (Fig. 72) hyaline, a faintly infuscate area below marginal and stigmal veins, and with linea calva interrupted, venation yellow-brown; hind wing hyaline; propodeum medially brown, laterally white; gaster mostly brown but dark brown dorsally from cercal plates to near apex, sides and venter white**;** ovipositor sheath yellow.

Head with polygonally reticulate sculpture and mesh size slightly less than that of one eye facet; ocelli forming an angle of about 35°; eye not quite reaching occipital margin, separated by much less than diameter of a facet; frontovertex subparallel-sided; scrobes shallow and U-shaped; antenna (Fig. 70) with scape about 2.4× as long as broad; funicle with F1–F4 smallest, subequal and transverse, F5 a little larger but transverse, F6 largest and quadrate, linear sensilla only on F6, clava 3-segmented, its apex more or less rounded but with a short slightly oblique truncation; mandible relatively broad with three subequal, apical teeth; palpal formula 2-2 (Fig. 71); notaular lines reaching about 0.6× across mesoscutum; fore wing venation and setation as in Fig. 72; ovipositor (Fig. 76) slightly exserted, about 5.2× as long as ovipositor sheath, second valvifer without subapical setae.

Relative measurements: HW 14, FV 4, FVL 8, POL 1.5, AOL 3, OOL 1, OCL 1, POD 1, AOD 1, EL 8, EW 6.5, MS 4, SL 6, SW 2.5, FWL 35, FWW 14, OL 10, GL 2, MT 11.

**Male.** Unknown.

##### Host.

Unknown.

##### Distribution.

China (Beijing, Hainan, Shanxi).

##### Etymology.

The species name ‘fusiformis’ is derived from the infuscate area of the fore wing.

##### Diagnosis.

Scape with both faces dark brown, blackish, dorsal margin narrowly pale yellow, extreme apex white and about 2.4× as long as broad; fore wing (Fig. 72) hyaline, a faintly infuscate area below marginal and stigmal veins.Using the key of Guerrieri and Noyes (2000), this species runs to couplet 10 and is similar to *Metaphycus ibericus* in having a uniformly weakly infuscate fore wing. It can be separated from the latter as follows: dorsal margin of scape pale orange, not marked brown medially and 2.4× as long as broad (in *ibericus*, dorsal margin of scpae marked brown medially and 3× as long as broad); ovipositor about as long as mid tibia (in *ibericus* with ovipositor about 0.8× as long as mid tibia).

#### Metaphycus xujiangi

Özdikmen, 2011

http://species-id.net/wiki/Metaphycus_xujiangi

Figs 77–83

Metaphycus tamakatakaigara Jiang 1982: 7: 182; Jiang 1986: (3):14. Misidentified.
Metaphycus ericeri Xu & Jiang 1990: 203. Holotype ♀, China, ZJU & SCU; Jiao and Zhao 1999: 166-171.
Metaphycus xujiangi Özdikmen, 2011: 802. Replacement name for *Metaphycus ericeri* Xu & Jiang nec Trjapitzin (1967).

##### Female.

**Figures 77–83.**
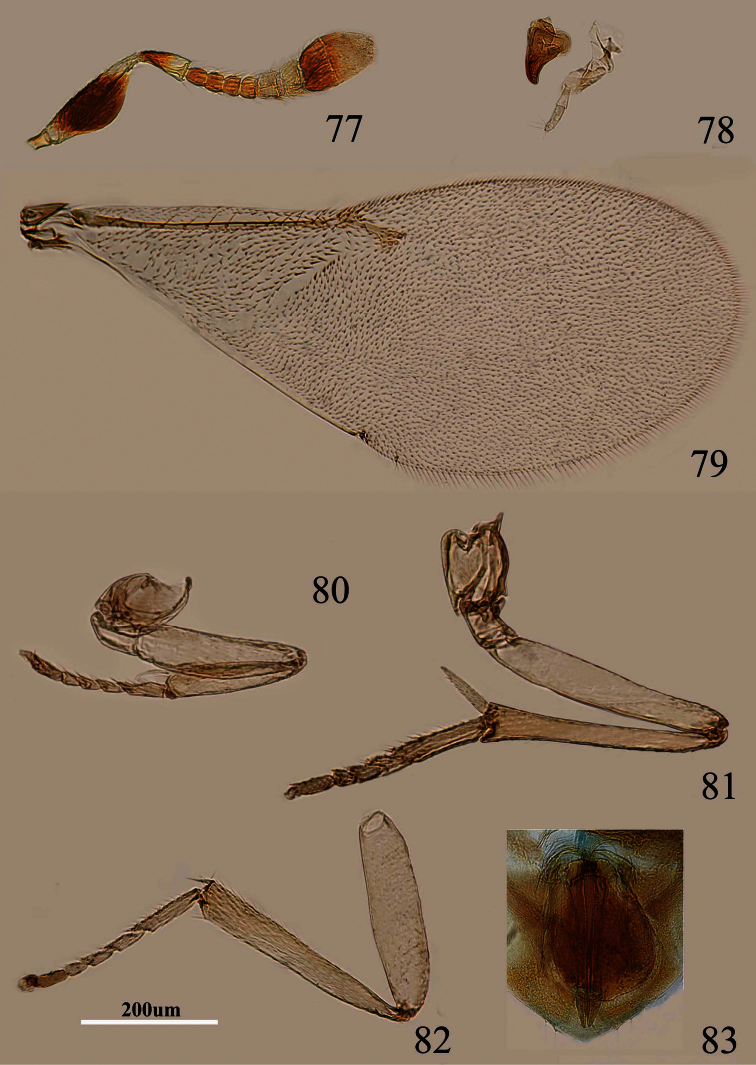
*Metaphycus xujiangi* Özdikmen. Female: **77** antenna **78** palpal formula and mandible **79** fore wing **80** fore leg **81** mid leg **82** hind leg **83** ovipositor.

Body length, including ovipositor about 1.1mm. Frontovertex orange to dark orange; orange in ocellar area, orange between occipital margin and posterior ocelli; immaculate from occiput to base of mandible; rest of head, except occiput, yellow-white; antenna (Fig. 77) with radicle yellow; scape with both faces blackish, only extreme apex and extreme distal yellow, dorsal margin black; pedicel dark brown in proximal half and apex white; F1–F4 brown, F5–F6 white, clava dark brown, becoming paler towards apex, apex white; occiput with dark brown area above occipital foramen, rest white; neck of pronotum dark brown, posterior margin translucent white, lateral spots relatively small and undistinct, rest white; dorsum of thorax orange; sides and posterior margin of mesoscutum and axillae bordered brown; setae translucent yellow, silvery in most lights; tegula white; metanotum pale brown; mesopleuron yellow; prosternum and mesosternum pale yellow; legs (Figs 80–82) mainly pale yellow; fore wing (Fig. 79) hyaline, and with linea calva interrupted, venation dark yellow; hind wing hyaline; propodeum medially dark orange; gaster dorsally pale brown, becoming paler towards apex, side and venter white; ovipositor sheath yellow.

Ocelli forming an angle of about 50°; eye not quite reaching occipital margin, separated by much less than diameter of one facet; frontovertex subparallel-sided, becoming slightly broader anteriorly from the narrowest point which is slightly in front of posterior ocelli; scrobes shallow and U-shaped; antenna with scape about 2–2.5× as long as broad; funicle with F1–F4 smallest, subequal, F4 transverse, F5 a little larger but transverse, F6 largest; clava 3-segmented, its apex more or less rounded and with a short slightly oblique truncation; mandible relatively broad with three subequal, apical teeth; palpal formula 2-2 (Fig. 78), notaular lines reaching about 0.6× across mesoscutum; fore wing venation and setation as in Fig. 79; ovipositor (Fig. 83) hardly exserted, length about 5.4× as long as ovipositor sheath.

Relative measurements: HW 15.5, FV 3, FVL 7,POL 2, AOL 3, OOL 1, OCL 1, POD 2, AOD 2, EL 9, EW 6, MS 4, SL 7, SW 3, FWL 45, FWW 21, HWL 30, HWW 6, OL 11, GL 2, MT 15.

##### Male.

(length 0.8–1.33mm). Thorax black-brown, ocellar area black-brown, antenna yellow-brown, clava solid and as long as F3 to F6. Digitus of genitalia apically with two hooks; aedeagus robust, length about 3× as long as broad. (Xu and Jiang 1990).

##### Host.

*Ericerus pela*.

##### Distribution.

China (Hunan, Sichuan, Yunnan).

##### Material examined.

China: 9♀♀, Sichuan, E’mei Mt., 1963.X, Coll. D. X Liao (IZCAS).

##### Diagnosis.

Antenna with radicle yellow; scape with both faces blackish, only extreme apex and extreme distal yellow, dorsal margin black, scape about 2–2.5× as long as broad; ovipositor hardly exserted, length about 5.4× as long as ovipositor sheath. Jiang (1982) misidentified this species as *Metaphycus tamakatakaigara*, and Xu and Jiang 1990 described it as a new species.*Metaphycusxujiangi* is very similar to *Metaphycus ericeri*. It can be separated from *Metaphycus ericeri* as follows: head is about 5× as broad as frontovertex, POD=POL and the ovipositor about 0.7× as long as mid tibia (in *Metaphycus ericeri*, the head is about 3.5× as broad as frontovertex, 2POD=POL and the ovipositor about 0.6× as long as mid tibia).

**Figures 84–86.**
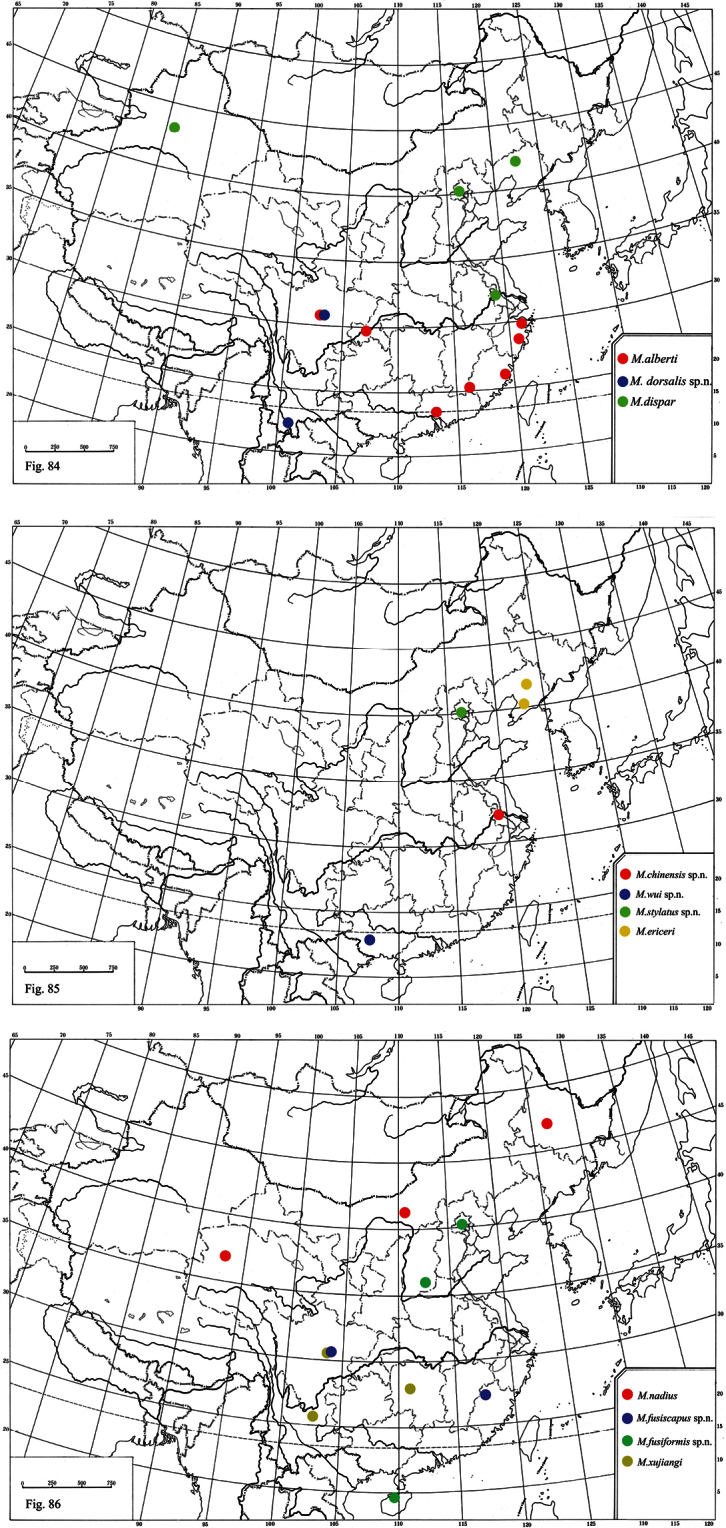
Distribution of*Metaphycus* spp. in China.

## Supplementary Material

XML Treatment for Metaphycus dorsalis

XML Treatment for Metaphycus alberti

XML Treatment for Metaphycus dispar

XML Treatment for Metaphycus chinensis

XML Treatment for Metaphycus wui

XML Treatment for Metaphycus stylatus

XML Treatment for Metaphycus ericeri

XML Treatment for Metaphycus nadius

XML Treatment for Metaphycus fusiscapus

XML Treatment for Metaphycus fusiformis

XML Treatment for Metaphycus xujiangi
